# Proinflammatory cytokines suppress nonsense-mediated RNA decay to impair regulated transcript isoform processing in pancreatic β cells

**DOI:** 10.3389/fendo.2024.1359147

**Published:** 2024-03-22

**Authors:** Seyed M. Ghiasi, Piero Marchetti, Lorenzo Piemonti, Jens H. Nielsen, Bo T. Porse, Thomas Mandrup-Poulsen, Guy A. Rutter

**Affiliations:** ^1^ Section of Cell Biology and Functional Genomics, Division of Diabetes, Endocrinology and Metabolism, Department of Metabolism, Digestion and Reproduction, Faculty of Medicine, Imperial College London, London, United Kingdom; ^2^ Department of Biomedical Sciences, University of Copenhagen, Copenhagen, Denmark; ^3^ Development and Aging Program, and Human Genetics Program, Sanford Burnham Prebys Medical Discovery Institute, La Jolla, United States; ^4^ Department of Clinical and Experimental Medicine, Islet Cell Laboratory, University of Pisa, Pisa, Italy; ^5^ Diabetes Research Institute, Istituto Di Ricovero e Cura a Carattere Scientifico (IRCCS) Ospedale San Raffaele, Milano, Italy; ^6^ Biotech Research and Innovation Centre (BRIC), University of Copenhagen, Copenhagen, Denmark; ^7^ The Finsen Laboratory, Rigshospitalet, Faculty of Health Sciences, University of Copenhagen, Copenhagen, Denmark; ^8^ Danish Stem Cell Center (DanStem) Faculty of Health Sciences, University of Copenhagen, Copenhagen, Denmark; ^9^ Centre Hospitalier de l'Université de Montréal (CHUM) Research Centre (CRCHUM) and Faculty of Medicine, University of Montreal, Montreal, QC, Canada; ^10^ Lee Kong Chian School of Medicine, Nanyang Technological University, Singapore, Singapore

**Keywords:** β-cells, insulin secretion, transcript, nonsense-mediated decay, RNA decay, RNA processing

## Abstract

**Introduction:**

Proinflammatory cytokines are implicated in pancreatic ß cell failure in type 1 and type 2 diabetes and are known to stimulate alternative RNA splicing and the expression of nonsense-mediated RNA decay (NMD) components. Here, we investigate whether cytokines regulate NMD activity and identify transcript isoforms targeted in ß cells.

**Methods:**

A luciferase-based NMD reporter transiently expressed in rat INS1(832/13), human-derived EndoC-ßH3, or dispersed human islet cells is used to examine the effect of proinflammatory cytokines (Cyt) on NMD activity. The gain- or loss-of-function of two key NMD components, UPF3B and UPF2, is used to reveal the effect of cytokines on cell viability and function. RNA-sequencing and siRNA-mediated silencing are deployed using standard techniques.

**Results:**

Cyt attenuate NMD activity in insulin-producing cell lines and primary human ß cells. These effects are found to involve ER stress and are associated with the downregulation of UPF3B. Increases or decreases in NMD activity achieved by UPF3B overexpression (OE) or UPF2 silencing raise or lower Cyt-induced cell death, respectively, in EndoC-ßH3 cells and are associated with decreased or increased insulin content, respectively. No effects of these manipulations are observed on glucose-stimulated insulin secretion. Transcriptomic analysis reveals that Cyt increases alternative splicing (AS)-induced exon skipping in the transcript isoforms, and this is potentiated by UPF2 silencing. Gene enrichment analysis identifies transcripts regulated by UPF2 silencing whose proteins are localized and/or functional in the extracellular matrix (ECM), including the serine protease inhibitor SERPINA1/α-1-antitrypsin, whose silencing sensitizes ß-cells to Cyt cytotoxicity. Cytokines suppress NMD activity via UPR signaling, potentially serving as a protective response against Cyt-induced NMD component expression.

**Conclusion:**

Our findings highlight the central importance of RNA turnover in ß cell responses to inflammatory stress.

## Introduction

Inflammatory and glucolipotoxic (GLT) stress causing β-cell failure and destruction *in vitro* differentially regulates hundreds of β-cell transcripts (1, 2). The upregulation of splicing factors and of proteins involved in pre-mRNA processing gives rise to alternative splicing (AS) events, which in turn deregulates the balance and turnover of transcript isoforms (3). Interestingly, most human mRNAs exhibit alternative splicing, but not all alternatively spliced transcripts are translated into functional proteins and are therefore targeted for degradation via the RNA decay pathways.

In addition to regulating the expression of normal transcripts, the human nonsense-mediated RNA decay (NMD) machinery functions to eliminate premature termination codon (PTC)-containing mRNAs, as reviewed extensively (4). Alternatively, spliced mRNA species and translation of dominant transcript isoforms vary in a cell-specific manner and depend on the capacity of cells to cope with damaged transcripts (5–7). A substantial number (i.e., around 35%, depending on tissue and physiological conditions) of alternatively spliced variants contain a PTC (4, 8, 9). Approximately 35% of the cytokine-regulated transcripts in human islets undergo alternative splicing (6), and Cyt profoundly upregulates NMD in rat and human insulin-producing cell lines and primary β cells, likely to handle the NMD load inferred by PTC-containing splice variants (4, 10, 11).

However, in addition to canonical NMD, in which all key NMD components function on target transcripts, a second branch of NMD is (in)dependently regulated in an autoregulatory feedback loop by its key factors, including UPF2 and UPF3 in a cell type-specific manner, as reviewed previously (4, 10).

In a previous study (11), we profiled the expressional level of NMD components and their regulation by cytokines and GLT in insulin-producing cells, but the NMD activity and its consequences for the β-cell transcriptome remained to be investigated. Here, using a luciferase-based NMD activity reporter, gain-/loss-of-function, and RNA-sequencing analyses in rodent and human β-cell systems, we measured NMD activity and explored its consequences for the function and viability of pancreatic β cells under normal conditions and inflammatory stress.

## Materials and methods

### Cell culture, human islet dispersion, and treatment

INS1(832/13) (12), EndoC-βH3 (13), or dispersed human islet cells were cultured and manipulated according to the protocols and procedures described in the **Supplementary Methods**.

### Luciferase-based NMD activity assay

One million cells were cotransfected with 650 ng of plasmid encoding either human *Haemoglobin-β* (*HBB*) wildtype (WT or PTC^−^) or with a PTC-containing mutation (NS39 or PTC^+^) fused with *Renilla* (*RLuc*), in brief, named PTC^−^ and PTC^+^, respectively. *Firefly* (*FLuc*) plasmid (14) was used as a transfection efficiency reference. *Renilla* and *Firefly* luminescence were measured by Dual-Luciferase Reporter Assay (Promega, Hampshire, England) (**Supplementary Methods**). *RLuc* signals were normalised to the *FLuc* control in both HBB NS39, in the following named HBB(PTC^+^), and HBBWT, in the following named HBB (PTC^−^), and NMD activity was calculated by dividing the *RLuc/FLuc*-HBB (PTC^−^) by the *RLuc/FLuc-*HBB (PTC^+^) (**Supplementary Figure S1A**) (14). Experiments where the control construct *RLuc/FLuc*-HBB(PTC^−^) was affected by cytokines were excluded, so that the resulting NMD activity only denotes the PTC-containing HBB(PTC^+^). The transfection efficiency was tested twice and resulted in an average of 80% in INS1 and EndoC-βH3 cells, as measured by FACS analysis of cells transfected with a GFP-expressing plasmid (**Supplementary Figures S1B, C**).

### Functional analysis of UPF3A/B overexpression

One million INS1(832/13) or EndoC-βH3 cells were transfected with 650 ng of plasmids encoding UPF3A, UPF3B, or UPF3BΔ42 (15), then simultaneously with NMD activity reporter plasmids (as above, 650 ng/million cells), recounted and seeded for Western blotting, glucose-stimulated insulin secretion (GSIS), viability, apoptosis (detailed below), and NMD activity assays in relevant plates and preincubated for 48 h before treatment with cytokines as explained in the **Supplementary Methods**.

### Lentiviral shRNA gene knockdown

GPIZ lentiviral shRNA particles directed against *UPF2*, *Upf3A*, or *Upf3B*, and a nonsilencing shRNA (NS) as a negative control were produced using the Trans-Lentiviral shRNA Packaging System in HEK293 cells (Horizon, Cambridge, England) according to the manufacturer’s protocol (**Supplementary Methods**).

### Apoptosis and cell viability assays

Apoptosis assays were performed in duplicate by detection of caspase-3 activity using a fluorometric (µM AMC) (or/colourimetric [µM PNA/min/mL] unless stated) assay kit (Cat No. APPA015-1KT/CASP3C-1KT, Sigma, London, England) according to the manufacturer’s protocols. Cell viability was measured by the Alamarblue assay (Cat No. DAL1025, LifeTechnologies, Renfrew, England) as previously described (11).

### Library preparation, RNA-sequencing, and data analysis

Using TRIZOL, 33 independent biological replicates of total RNA from the NS control and/or UPF2 KD EndoC-βH3 cells exposed to cytokines, GLT, or PBS (i.e., *N* = 6 of each PBS-/or cytokine-exposed NS control and UPF2 KD, and *N* = 4/*N* = 5 of GLT-exposed NS control/UPF2 KD, respectively) were extracted, treated with DNase, and precipitated with isopropanol (**Supplementary Methods**). Following the manufacturer’s recommendations, 1 µg of total RNA/per isolate was used as input for the generation of sequencing libraries using NEBNext^®^Ultra-TM RNA Library-Prep (Cat No. E7770, NEB, Ipswich, MA, USA) (**Supplementary Methods**). The RNA-seq raw data underwent quality control and were mapped to the human reference genome (16) and analysed using the bioinformatic pipeline described in the **Supplementary Methods**.

### cDNA synthesis and RT-qPCR

Purified total RNA (500 ng) was used for cDNA synthesis with the SuperScript™ (Cat No. 11904018, LifeTechnologies). Real-time reverse transcriptase-quantitative PCR (RT-qPCR) was performed on 12 ng cDNA with SybrGreen PCR Master Mix (LifeTechnologies) and specific primers (**Supplementary Table S2**) and run in an ABI Real-Time PCR Machine (Applied Biosystems, ThermoFisher Scientific, Oxford, England). The raw data was analysed through −ΔCt as described in the **Supplementary Methods**.

### Western blotting

Western blotting was performed using antibodies against alpha-tubulin (1:2,000) (Cat No. T5168, Sigma), UPF2 (1:1,000) (Cat No. PA5-77128, LifeTechnologies), UPF3A (1:1,000) (Cat No. PA5-41904, LifeTechnologies), UPF3B (1:1,000) (Cat No. PB9843, Boster-Bio, Pleasanton, CA, USA) and α-1-antitrypsin (1:1,000) (Cat No. TA500375, LifeTechnologies) as described in the **Supplementary Methods** (17).

### Glucose-stimulated insulin secretion

In 12-well plates (Cat No. 150200, Nunc, Buckingham, England), 300,000 INS1(832/13) or EndoC-βH3 cells were cultured and preincubated for 2 days. GSIS was carried out using Krebs–Ringer buffer containing 2 mM or 17 mM glucose, as described (11, 18).

### Insulin assay

Insulin concentration (ng/mL or pM) was measured using a rat insulin ultrasensitive ELISA kit (Cat No. 62IN2PEG, Cisbio, Cambridge, England) or human insulin ELISA Kit (Cat No. 90095, CrystalChem, IL, USA), respectively, according to the manufacturer’s protocol.

### Statistical analysis

Data are presented as means ± SEM. Statistical analysis was carried out on raw data in cases where figures gave normalised data. Group comparisons were carried out by two- or one-way ANOVA as appropriate. Significant ANOVAs were followed by a *post-hoc* paired Student’s *t*-test with Bonferroni correction using GraphPad Prism 6.0 (La Jolla, CA, USA). A paired *t*-test was chosen to normalise for interpassage variability in outcome parameters. Since the experimental conditions did not allow sequential sampling from the same cell culture, parallel control and interventional plate wells were considered to be paired observations and analysed accordingly statistically. If the *post-hoc* paired *t*-test did not reveal a carrying statistical difference by ANOVA, individual paired *t*-tests were performed and corrected for multiple comparisons. Bonferroni-corrected *p*-values ≤ 0.05 were considered significant and ≤ 0.10 a trend.

## Results

### Cytokines suppress NMD activity in β cells

We previously reported that cytokines and glucolipotoxicity differentially up- or downregulate NMD component transcripts in pancreatic β cells (11). However, whether this regulation leads to increased NMD *activity* remains to be elucidated. Here, we used a luciferase-based NMD reporter (**Supplementary Figure S1A**) (14) to examine NMD activity in rat INS1(832/13), human insulin-producing EndoC-βH3 cells, and primary human islets. Luciferase activity analysis showed that cytokines (Cyt; 150 pg/mL IL-1β + 0.1 ng/mL IFN-γ + 0.1 ng/mL TNF-α) significantly suppressed NMD activity by nearly 50% after 18 h, but not 6 h, of exposure in INS1(832/13) cells [**Supplementary Figures S2A**(i, iii)].

**Figure 1:**
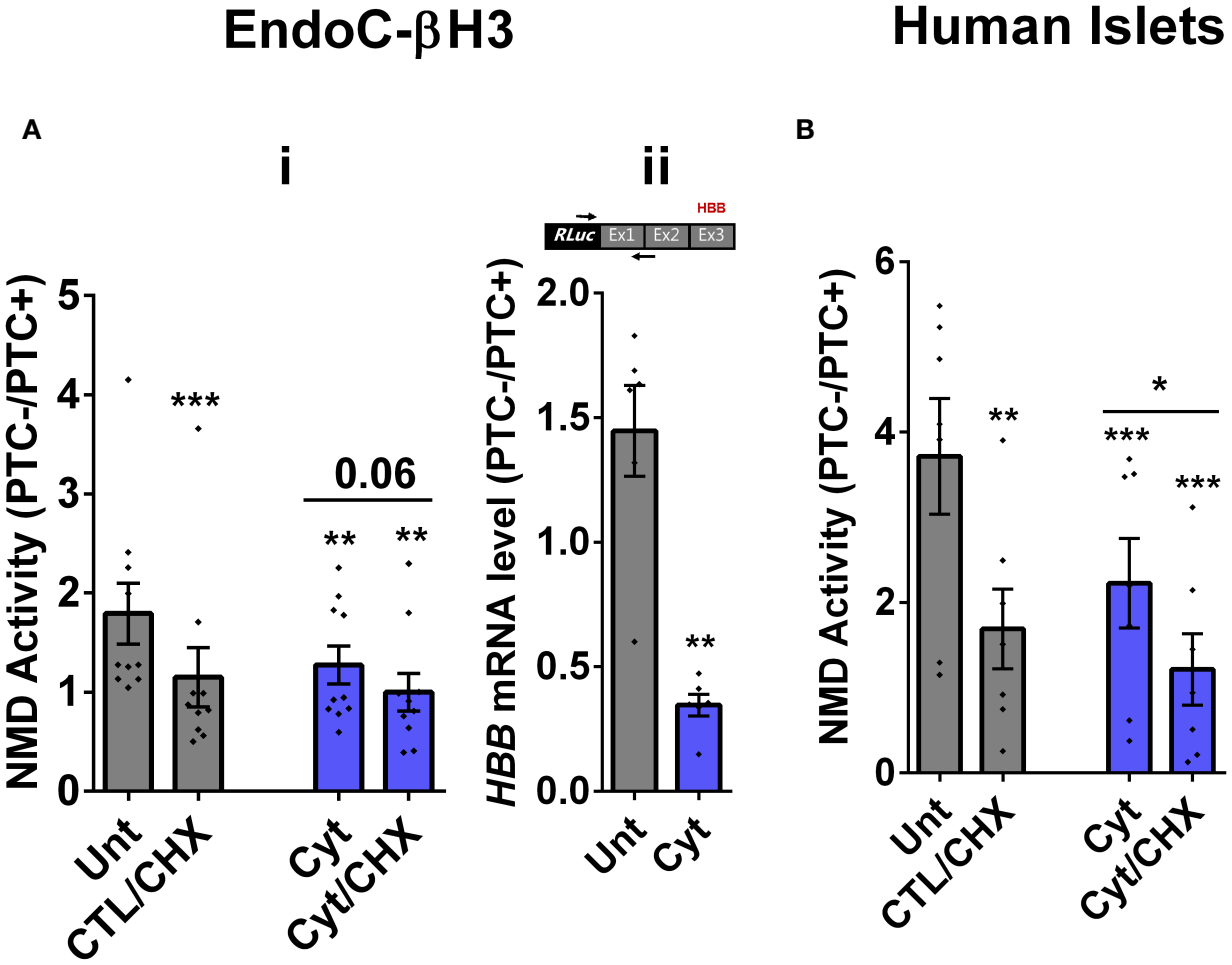
Cytokines suppress NMD activity in β cells. **(A, B)** EndoC-βH3 cells **(A)** and dispersed human islet cells **(B)** were cotransfected with *Renilla*-HBB(WTnamed PTC^−^) and or *Renilla*-HBB (NS39 (named PTC^+^)) and the *Firefly* plasmids and exposed to cytokine combination and or PBS as untreated (Unt) simultaneously with or without cycloheximide (CHX) as a positive control for inhibited NMD activity. Luciferase activity was measured in the lysate of the transfected EndoC-βH3 cells **(A ** (i))and dispersed human islet cells **(B)** exposed to cytokine combination (Cyt; 3 ng/mL IL-1β + 10 ng/mL IFN-γ + 10 ng/mL TNF-α) for 18 (h) **(A **(ii)) mRNA level of *Renilla-HBB*-fused gene and the *Firefly* gene in the transfected EndoC-βH3 cells was quantified by RT-qPCR using specific primers extending the junction of exons 1 and 2 of the *HBB* gene and the *Renilla* gene, or only the *Firefly* gene, and normalised to actin and tubulin, respectively. The data are means ± SEM of *N* = 6. The symbol “^*^” indicates the Bonferroni-corrected paired *t*-test values of treated versus untreated (Unt) **(A, B)** or otherwise, cytokines (Cyt) that are designated by a line on top of the bars **(A**, **B)**: ^*^≤ 0.05; ^**^≤ 0.01; ^***^≤ 0.001; ^****^≤ 0.0001. ns, nonsignificant; HBB, *haemoglobin-β*; PTC, premature termination codon; RLuc, *Renilla* luciferase; Ex, exon.

We then tested the effects of cytokines on EndoC-βH3 cells and dispersed human islet cells. Cytokines [2.5 ng/mL IL-1β + 10 ng/mL TNF-α + 10 ng/mL IFN-γ, chosen from dose–response experiment shown in **Supplementary Figure S2B** (i)] attenuated NMD activity by 30% (*p =* 0.009, *n* = 6) and 40% (*p =* 0.0006, *n* = 6) after 18 h of exposure to EndoC-βH3 cells (**Figure 1A**i) and dispersed human islet cells (**Figure 1B**), respectively. Cyt increased the luciferase signal (*RLuc/FLuc*) from the HBB(PTC^-^) (**Supplementary Figures S2B**(ii), **C**) but not from the HBB(PTC^-^) [**Supplementary Figures 2B**(v), **C** (ii)] in both cell models, confirming that the NMD substrate HBB(PTC^+^) was restored due to NMD activity attenuation by Cyt.

We next examined whether cytokine-mediated suppression of NMD was consistent with an accumulation of HBB(PTC^+^) *transcripts*. For this, we used a forward and reverse primer set to amplify the *Renilla* gene and the junction of exons 1 and 2 (i.e., ensuring amplification of mature transcripts only), respectively. RT-qPCR analysis demonstrated that cytokines caused significant upregulation of HBB(PTC^+^) but not HBB(PTC^−^) mRNA levels, rendering a significant reduction of the relative PTC^−^/PTC^+^ mRNA levels in INS1(832/13) (*p =* 0.008) [**Supplementary Figure S2A**(v, vi)] and EndoC-βH3 (*p =* 0.001) cells [**Figure 1A**ii; **Supplementary Figure 2B**(iv)], which verified the suppressive effect of cytokines on NMD activity.

We also examined the effect of 25 mM glucose or GLT conditions on NMD activity. Unfortunately, these conditions significantly affected the luciferase signal (*RLuc/FLuc*) from the HBB(PTC^−^) control, preventing further study of the effects of metabolic stressors on NMD activity.

Taken together, these results show that cytokines suppress the activity of the NMD in a range of insulin-secreting cell types.

### Cytokine-induced suppression of NMD activity in β cells is ER stress-dependent

**Figure 2:**
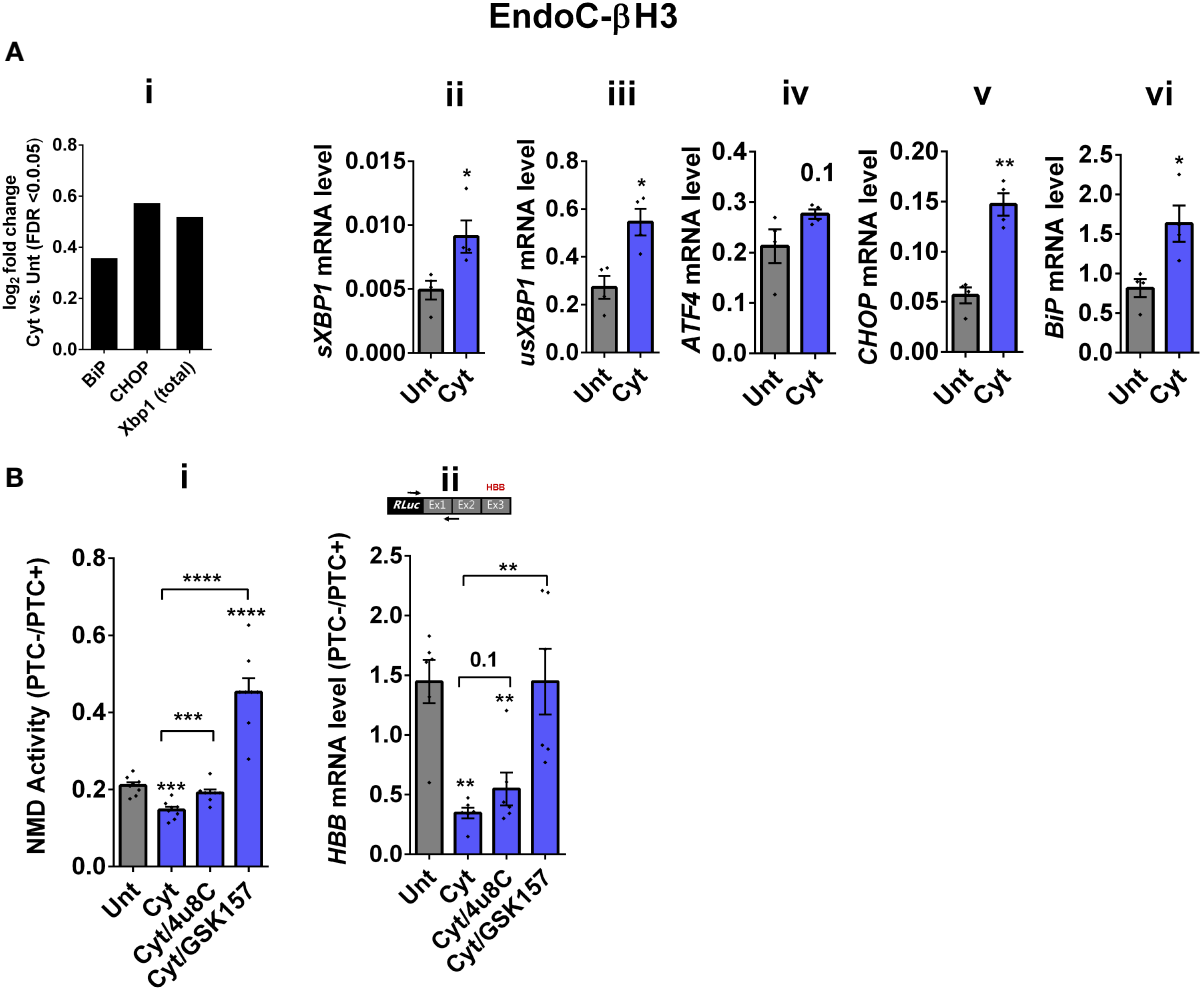
Cytokine-induced suppression of NMD activity in β cells is ER stress-dependent. **(A)** mRNA levels of ER stress markers in EndoC-βH3 cells exposed to cytokine combination (Cyt; 3 ng/mL IL-1β + 10 ng/mL IFN-γ + 10 ng/mL TNF-α) for 18 h were quantified by RNA sequencing (**A**(i)) with false discovery rate (FDR) < 0.05 presented as logarithmic fold change of the cytokine (Cyt) treatment versus control (untreated) and RT-qPCR (ii–v), which was normalised to tubulin mRNA. **(B)** EndoC-βH3 cells were cotransfected with *Renilla*-HBB(PTC^−^) and/or *Renilla*-HBB(PTC^+^) and the *Firefly* plasmid and exposed to PBS as untreated (Unt), cytokine combination (Cyt; 3 ng/mL IL-1β + 10 ng/mL IFN-γ+ 10 ng/mL TNF-α) alone, and/or simultaneously with 16 µM of 4μ8C, an endoribonuclease inhibitor of IRE1α, and/or 8 µM of GSK2656157 (GSK157), PERK inhibitor for 18 (h) (**B**(i)) Luciferase activity was measured in the lysate of EndoC-βH3 cells transfected with *Renilla*-HBB(PTC^−^) and or *Renilla*-HBB(PTC^+^) and the *Firefly* plasmid exposed to PBS as untreated (Unt) or given conditions, and represented as NMD activity calculated by dividing luciferase activity of HBB(PTC^−^)/HBB(PTC^+^) as explained in the Methods. (ii) mRNA level of *Renilla-HBB*-fused gene and *Firefly* gene in the EndoC-βH3 cells was quantified by RT-qPCR using specific primers extending the junction of exons 1 and 2 of the *HBB* gene and the *Renilla* gene, or only *Firefly* gene, and normalised to tubulin. The data are means ± SEM of *N* = 6. The symbol “^*^” indicates the Bonferroni-corrected paired *t*-test values of treated versus untreated (Unt) **(A, B)** or, otherwise, cytokines (Cyt) that are designated by a line on top of the bars **(B)**: ^*^≤ 0.05; ^**^≤ 0.01; ^***^≤ 0.001; ^****^≤ 0.0001. FDR, false discovery rate; RLuc, *Renilla* luciferase; Ex, exon.

Whereas NMD degrades unfolded protein response (UPR)-induced transcripts in compensated ER stress, NMD is suppressed in response to pronounced endoplasmic reticulum (ER) stress to allow a full-blown UPR (19, 20). Cytokines induce a robust ER stress in pancreatic β cells, largely via nuclear factor-κB (NF-κB) activation and production of nitroxidative species that inhibit the smooth endoplasmic reticulum Ca^2+^ ATPase (SERCA) 2B pump, leading to ER calcium depletion (11, 17). We have previously shown that chemical inhibition of inducible nitric oxide synthase (iNOS) alleviated ER stress and normalised cytokine-mediated regulation of NMD components in INS1 cells (11). Therefore, we asked if cytokine-mediated reduction of NMD activity was dependent on an ER stress response in β cells. We first demonstrate that thapsigargin (TG), a noncompetitive inhibitor of SERCA (21) and ER stress inducer (22), inhibited NMD activity by 50% in EndoC-βH3 cells, as measured by luciferase assay [**Supplementary Figures S3A**(i, ii)]. Compared to untreated EndoC-βH3 cells (Unt), cytokines significantly augmented the increase in mRNA levels encoding the ER stress markers BiP, Xbp1, and Chop (FDR < 0.05) measured by RNA-sequencing analysis (**Figure 2A**i) and later verified by RT-qPCR examination (**Figure 2A**ii–vi). Finally, compared with Unt, cytokines significantly decreased the NMD activity by 30%, and this effect was counteracted by the protein kinase R-like endoplasmic reticulum kinase (PERK) phosphorylation inhibitor GSK157 (8 µM) and by the inositol-requiring enzyme 1 (IRE1α) endoribonuclease inhibitor 4µ8C (16 µM) in EndoC-βH3 cells [**Figure 2B**i; **Supplementary Figures S3B, C**(i, ii)]. RT-qPCR analysis of the relative PTC^−^/PTC^+^ mRNA levels in EndoC-βH3 cells confirmed the NMD activity data [**Figure 2B**ii, **Supplementary Figure S3C**(iii)].

Taken together, these results demonstrate that inhibition of UPR antagonises the cytokine-mediated reduction of NMD activity in EndoC-βH3, indicating that cytokine-mediated inhibition of NMD activity is UPR-dependent.

### Cytokine-induced suppression of NMD activity is associated with UPF3B downregulation and is attenuated by UPF3 overexpression in β cells

**Figure 3:**
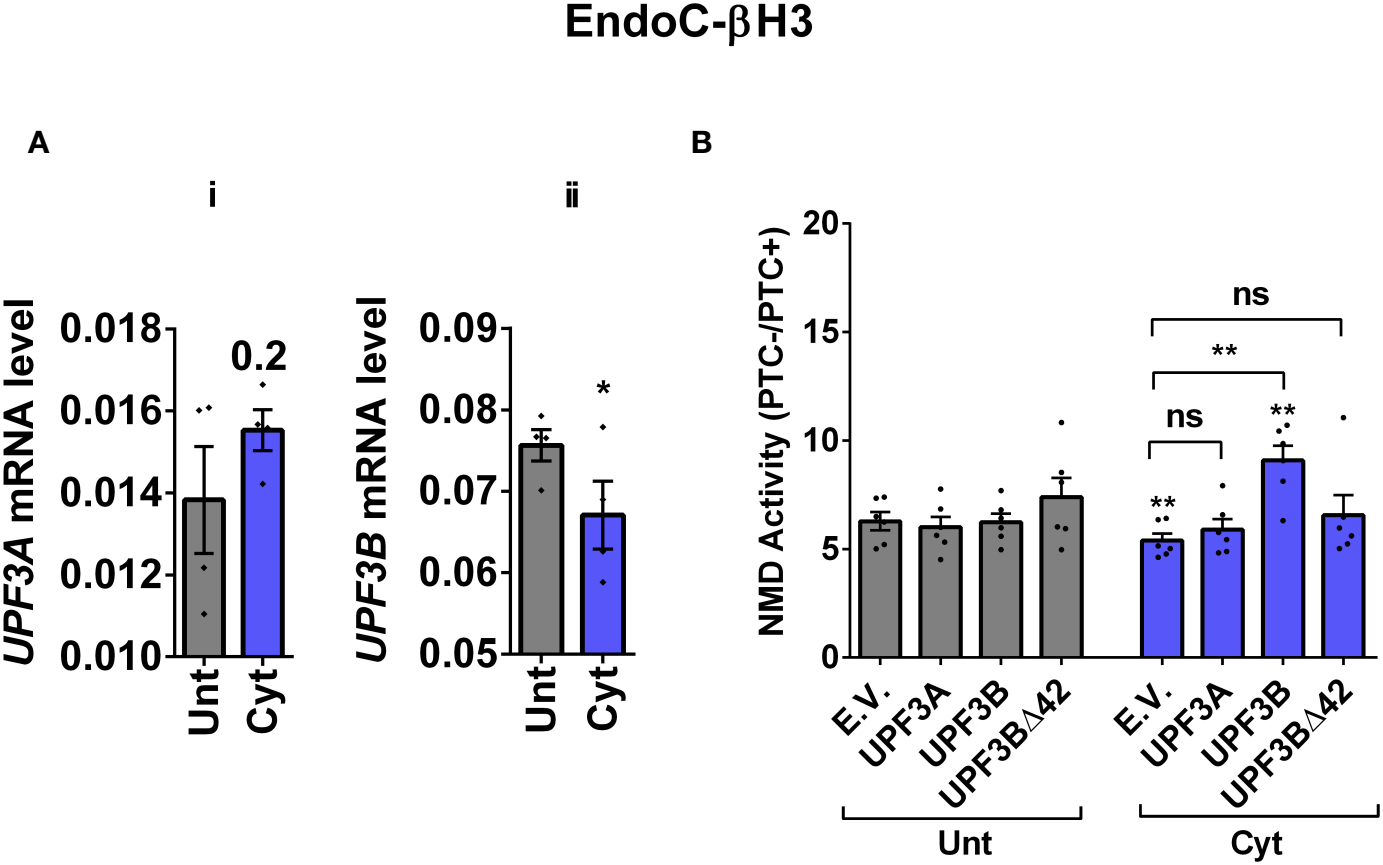
Cytokine-induced suppression of NMD activity is associated with UPF3B downregulation and attenuated by UPF3 overexpression in β cells. EndoC-βH3 cells were cotransfected with empty vector (E.V.), UPF3A, UPF3B, and/or UPF3BΔ42 (dominant negative of UPF3B) plasmids, and then with *Renilla*-HBB(PTC^−^) and or *Renilla*-HBB(PTC^+^), along with the *Firefly* plasmid and exposed to cytokine combination (Cyt; 3 ng/mL IL-1β + 10 ng/mL IFN-γ + 10 ng/mL TNF-α) for 18 (h) **(A**(i, ii)**)** mRNA level of *Upf3A* and *Upf3B* genes in EndoC-βH3 cells was quantified by RT-qPCR and normalised to tubulin mRNAs. **(B)** Luciferase activity was measured in the lysate of the transfected cells and represented as NMD activity calculated by dividing luciferase activity of HBB(PTC^−^)/HBB(PTC^+^) as explained in the Methods. The overexpression of UPF3A and UPF3B proteins was examined by Western blot analysis [**Supplementary Figure 4B**(i)]. The data are means ± SEM of *N* = 6. The symbol “^*^” indicates the Bonferroni-corrected paired *t*-test values of treated versus untreated E.V. (Unt) **(A, B)** or, otherwise, cytokine (Cyt)-treated E.V. that is designated by a line on top of the bars **(B)**: ^*^≤ 0.05; ^**^≤ 0.01. ns, nonsignificant.

Since we observed in our previous study that cytokine-induced ER stress downregulated UPF3B expression in human and rodent β cells, as recovering nitroxidative-driven ER stress using the inducible nitric oxide synthase (iNOS) inhibitor *N*-methyl-l-arginine (NMA) (11), since transcripts encoding UPR components are NMD targets and have been shown to be stabilised by UPF3A/B depletion (19), and since UPF3B is a NMD activator in mammalian cells (23), which led to the proposal of UPF3-dependent and UPF3-independent branches of the NMD pathway (4, 10, 24). We reasoned that UPF3 regulated NMD activity in β cells. We therefore first measured the UPF3A/B expression level and then investigated the functional impact of overexpressing UPF3A/B on cytokine-mediated suppression of NMD activity in β cells. RT-qPCR examination showed that cytokines significantly downregulated UPF3B mRNA levels after 18 h in both EndoC-βH3 (**Figures 3A**i, ii) and INS1(832/13) [**Supplementary Figures S4A**(i, ii)] as previously reported (11). Immunoblot analysis verified overexpression of UPF3A, UPF3B, and the UPF3B dominant negative UPF3BΔ42 in both EndoC-βH3 [**Supplementary Figure S4B**(i)] and INS1(832/13) [**Supplementary Figure S4C**(i)]. Cytokines reduced NMD activity, and overexpression of UPF3B significantly attenuated this reduction in EndoC-βH3 [**Figure 3B**; **Supplementary Figure S4B**(ii)] and to a lesser extent in INS1(832/13) [**Supplementary Figures S4C**(ii, iii)]. Neither UPF3A nor UPF3BΔ42 overexpression counteracted cytokine-attenuated NMD activity.

This result suggests that cytokines reduce NMD activity in β cells through downregulation of UPF3B expression.

### UPF3 overexpression deteriorates cell viability and reduces insulin content but not secretion in EndoC-βH3 cells

**Figure 4:**
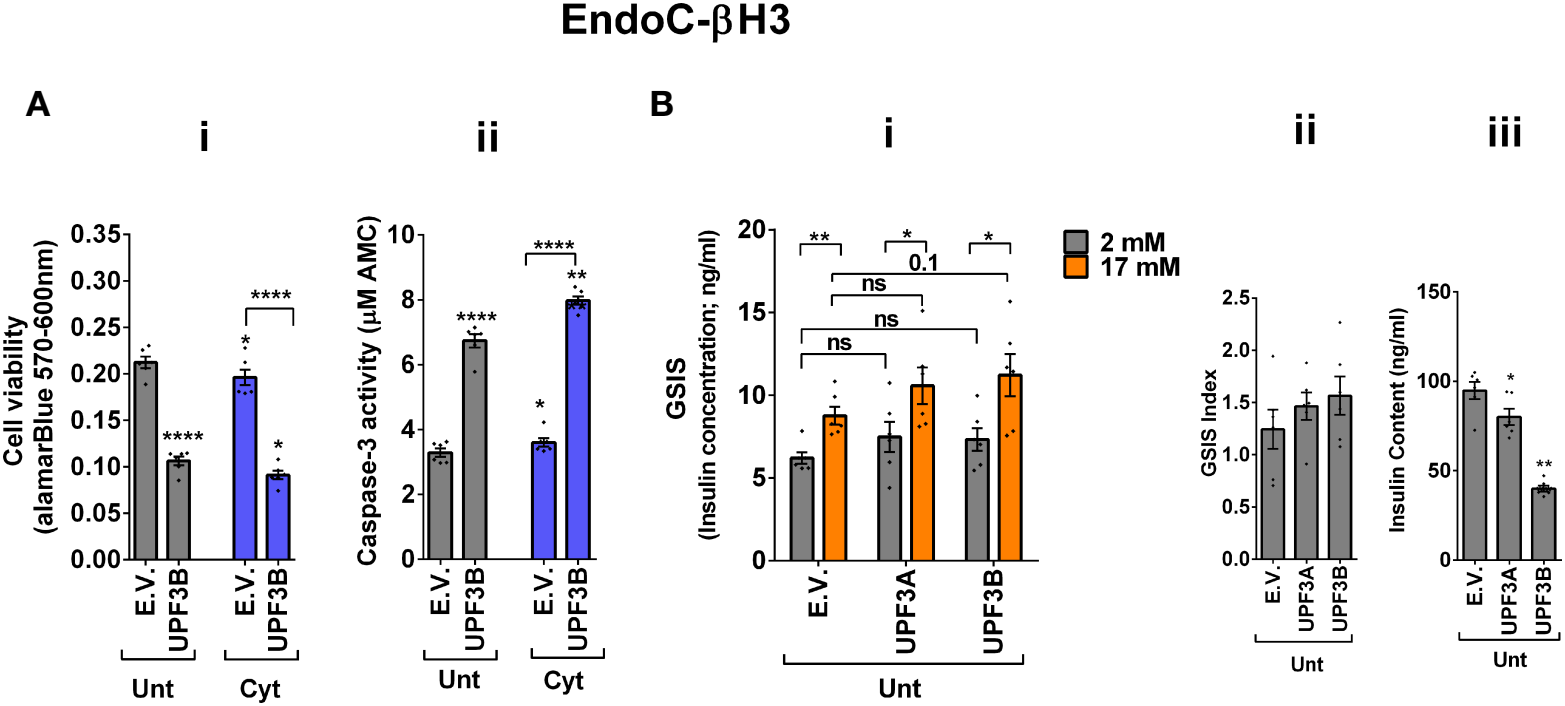
UPF3 overexpression deteriorates cell viability and reduces insulin content but not secretion in EndoC-βH3 cells. EndoC-βH3 cells were cotransfected with empty vector (E.V.), UPF3A, and/or UPF3B plasmids and exposed to cytokine combination (Cyt; 3 ng/mL IL-1β + 10 ng/mL IFN-γ + 10 ng/mL TNF-α) for 3 days. **(A)** Cell viability was measured by Alamarblue (i) and caspase-3 activity (ii) assays (*N* = 6). **(B)** Glucose-stimulated insulin secretion (GSIS) (i) and insulin contents (iii) were investigated in the transfected EndoC-βH3 cells. Insulin concentration (ng/mL) was measured by insulin ultrasensitive assay (*N* = 6). GSIS index (ii) was calculated by dividing the insulin concentration measured in the treatments at 17 mM by 2 mM of glucose. The data are means ± SEM of *N* = 6. The symbol “^*^” indicates the Bonferroni-corrected paired *t*-test values of treated versus untreated E.V. (Unt) or, otherwise, cytokine (Cyt)-treated E.V. that is designated by a line on top of the bars **(A)** or the Bonferroni-corrected paired *t*-test values of the corresponding low versus high glucose, that is otherwise designated by lines on top of the bars **(B**(i)**)**: ^*^≤ 0.05; ^**^≤ 0.01; ^****^≤ 0.0001. ns, nonsignificant.

The above findings provide evidence that the UPF3-dependent branch of NMD is involved in cytokine-mediated suppression of NMD activity. Therefore, we next investigated the impact of UPF3A/B overexpression on cytokine-induced cell death and insulin secretion. While UPF3A or UPF3B overexpression increased basal cell death, it also exacerbated the cytokine-induced apoptosis in EndoC-βH3 cells (**Figures 4A**i, ii). In INS1(832/13) cells, neither UPF3A nor UPF3B overexpression changed cell viability in the absence of Cyt exposure, but UPF3B overexpression significantly aggravated cytokine-induced cell death as measured by Alamarblue and caspase-3 activity assays [**Supplementary Figures S5A**(i, ii)]. Therefore, we next explored the impact of UPF3A or UPF3B deficiency on β-cell viability. Lentiviral shRNA-mediated knockdown of UPF3A and/or UPF3B [**Supplementary Figures S5B**(i, ii)] significantly reduced basal INS1(832/13) cell viability (**Supplementary Figure S5C**(i–iv). Taken together, these data indicate that genetic manipulations of UPF3A/B could possibly be detrimental to the β-cell viability.

In contrast to Cyt, which downregulates UPF3B in INS1 (11), EndoC-βH3 (**Figure 3A**ii), and INS1(832/13) cells [**Supplementary Figure S4A**(ii)], GLT does not downregulate UPF3B (11).We therefore explored the effect of UPF3 deficiency on glucolipotoxicity-induced cell death in β cells. Measurements of caspase-3 activity demonstrated that both UPF3A and UPF3B knockdown rendered a slight but significant protection against 24 h glucolipotoxicity in INS1(832/13) cells in comparison with untreated cells [**Supplementary Figure S5D**(i, ii)]. Furthermore, treatment with the NMD activator Tranilast dose-dependently sensitised to glucolipotoxicity-, but not cytokine-induced, EndoC-βH3 cell death, measured by caspase-3 activity assay [**Supplementary Figure S4E**(i, ii)]. Neither UPF3A nor UPF3B overexpression affected GSIS in EndoC-βH3 (**Figure 4B**i, ii) or INS1(832/13) [**Supplementary Figure S5F**(I, ii)] cells. Nonetheless, UPF3B overexpression profoundly lowered insulin content in EndoC-βH3 cells (**Figure 4B**iii). In contrast, knockdown of UPF3A or UPF3B significantly decreased the stimulatory index, as well as provoking a substantial increase in insulin content in control INS1(832/13) cells [**Supplementary Figures S5G**(ii, iii), **H** (ii and iii)], without altering ins1 or ins2 mRNA expression [**Supplementary Figures S5G** (iv, v), **H** (iv, v)].

Taken together, these findings reveal that UPF3 overexpression induces basal cell death and exacerbates cytokine-mediated toxicity in β cells.

### UPF2 knockdown potentiates cytokine suppression of NMD activity and slightly alleviates cytokine toxicity for cell viability and insulin content in EndoC-βH3 cells

**Figure 5:**
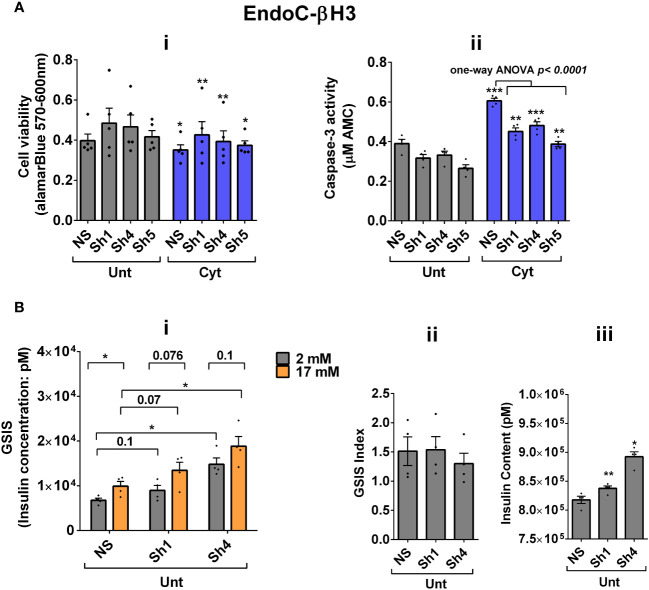
UPF2 knockdown potentiates cytokine suppression of NMD activity and slightly alleviates cytokine toxicity for cell viability and insulin content in EndoC-βH3 cells. EndoC-βH3 cell lines with the most efficient stable knockdown (KD) of UPF2 (three shRNAs; Sh1, Sh4, and Sh5) and nonsilencing shRNA control (NS) were cotransfected with *Renilla*-HBB(PTC^−^) and or *Renilla*-HBB(PTC^+^) and the *Firefly* plasmids and exposed to PBS as untreated (Unt) and or cytokine combination (Cyt; 3 ng/mL IL-1β + 10 ng/mL IFN-γ+10 ng/mL TNF-α). **(A)** Cell viability was measured by Alamarblue (i) and caspase-3 activity (ii) assays (*N* = 6). **(B)** Glucose-stimulated insulin secretion (GSIS) (i) and insulin contents (iii) were investigated in the UPF2 KD EndoC-βH3 cells. Insulin concentration (pM) was measured by human insulin ELISA (*N* = 6). GSIS index (ii) was calculated by dividing insulin concentration measured in the treatments of 17 mM by 2 mM glucose. The data are means ± SEM. The symbol “^*^” indicates the Bonferroni-corrected paired *t*-test values of treated versus untreated (Unt) NS control **(A**, **B)**, otherwise designated by a line on top of the bars, or the Bonferroni-corrected paired *t*-test values of corresponding low versus high glucose, that is, otherwise designated by lines on the top of the bars **(B**(i)**)**. ^*^≤ 0.05; ^**^≤ 0.01; ^***^≤ 0.001.

Next, we investigated the effect of UPF2 deficiency on the viability and insulin secretion of β cells because (i) UPF3A and UPF3B are involved in regulating UPF2, a key core NMD activator in mammalian cells (25, 26) by sequestering away from and bridging the exon-junction complex (EJC) with UPF1 and UPF2, respectively, leading to the NMD activation (23), and genome-wide association (GWAS) data reveal that the *UPF2* variant rs145580445 is significantly associated with type 2 diabetes risk (7). We, therefore, knocked down the *UPF2* gene in EndoC-βH3 cells using RNA interference and chose the three cell lines in which UPF2 was most efficiently knocked down (KD) [**Supplementary Figures S6A**(i, ii)]. Examination of NMD activity using the luciferase-based NMD reporter revealed that UPF2 KD profoundly reduced NMD activity in untreated and cytokine-treated EndoC-βH3 cells [**Supplementary Figures S6B**(i, ii)]. Compared with NS control, UPF2 KD slightly but significantly prevented cytokine-induced cell death (**Figures 5A**i, ii). UPF2 KD had no effect on the GSIS but significantly increased insulin content (**Figures 5B**i–iii).

These data indicate that UPF2 plays a crucial role in cytokine-induced β-cell apoptosis. In addition, the increase in insulin content in UPF2-deficient EndoCβH3 cells implies that insulin transcripts could possibly be targets of the UPF2-dependent NMD pathway branch.

### UPF2 knockdown differentially affects cytokine- and glucolipotoxicity-mediated deregulation of EndoC-βH3 transcripts

Consistent with our observations above (**Figure 5**), we previously reported (11) that the deficiency of SMG6, an endoribonuclease and a key effector of NMD, rendered protection against cytokine-induced cell death and was associated with increased insulin content. Therefore, we aimed to identify potential NMD target transcripts by using RNA-sequencing to assess the transcriptome of cytokine- or PBS-treated EndoC-βH3 cells stably transfected with a nonsilencing shRNA (NS) or the specific shRNA (shRNA-1 named U1) against *UPF2*. Due to the differential effects of Cyt and GLT on UPF3 expression cf. above, we also performed RNA-sequencing after UPF2 KD versus NS control EndoC-βH3 exposed to GLT compared with PBS-treated.

The RNA-seq datasets from either UPF2 KD or NS control EndoC-βH3 cell lines exposed to cytokines and or GLT were dimensionally reduced by principal component analysis (PCA) into two main principal components, PC1 and PC2 (*p* < 0.05). The PCA of the NS control EndoC-βH3 cells demonstrated a high similarity between the biological replicates, a small within-group variance, and a distinct clustering of the untreated cytokine and GLT groups [**Supplementary Figures S7A**(i, ii)]. Pearson’s correlation (*p* < 0.05) between samples justified the clustering of biological replicates of cytokines, GLT, and untreated conditions [**Supplementary Figures S7B**(i, ii)]. PCA revealed that the UPF2 knockdown increased the majority of variance in transcript isoforms of the cell, which led to patterns that visually dispersed biological replicates from the cytokine-exposed isolates, while decreasing the variances from the GLT and untreated biological replicates, which clustered them together.

**Figure 6:**
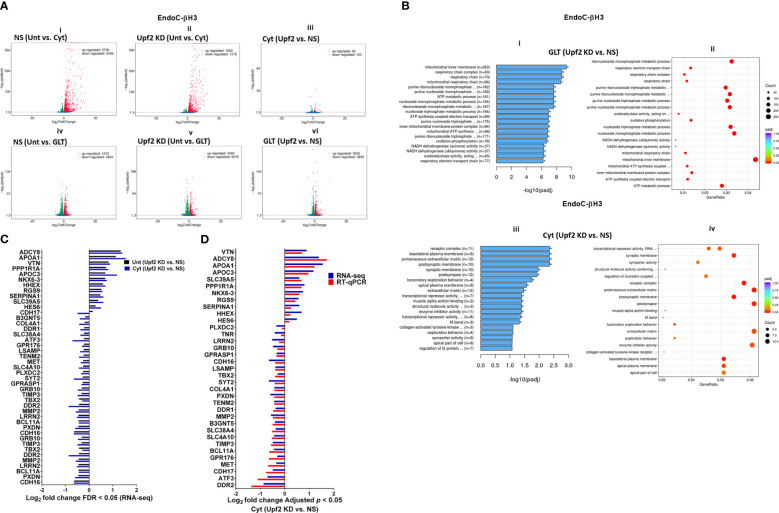
UPF2 knockdown differentially affects cytokine- and glucolipotoxicity-mediated deregulation of EndoC-βH3 transcripts. EndoC-βH3 cell lines knocked down for UPF2 (shRNA-1 named U1) or nonsilencing shRNA control (NS) were exposed to PBS as untreated (Unt), cytokine combination (Cyt; 3 ng/mL IL-1β + 10 ng/mL IFN-γ + 10 ng/mL TNF-α), and/or glucolipotoxicity (GLT; 0.5 mM palmitate +25 mM glucose). Total RNA was extracted from the treated cells, and cDNA library was made and sequenced using the Hiseq platform as explained in the Methods. In total, 33 RNA-seq datasets from NS/CTL (*N* = 6), NS/Cyt (*N* = 6), NS/GLT (*N* = 4), U1/CTL (*N* = 6), U1/Cyt (*N* = 6) and U1/GLT (*N* = 5) were analysed through the pipeline described in the **Supplementary Methods**. **(A)** Volcano plot of the number of transcripts (FDR < 0.05) regulated by Cyt or GLT versus untreated in the NS or UPF2 KD (U1) cell lines. **(B)** Top enriched pathways (i, iii) and the number of transcripts (ii, iv) regulated by Cyt and/or GLT in the UPF2 KD (FDR < 0.0.5). Enrichment is shown as log10 (adjusted *p*-value < 0.05). **(C)** Top GEA-identified transcripts regulated by Cyt compared to untreated in UPF2 KD versus NS control EndoC-βH3 cells. The expression level is shown as log2 (adjusted *p*-value < 0.05). **(D)** RT-qPCR verification of the identified transcripts regulated by Cyt in UPF2 KD versus NS control EndoC-βH3 cells. The expression level is shown as log2 (adjusted *p*-value < 0.05).

A Venn diagram of the RNA-seq datasets demonstrated an approximate total of 14,000 commonly expressed transcripts (FDR < 0.05) and those differentially expressed transcripts regulated by PBS (i.e., untreated control), cytokines, or GLT [**Supplementary Figures S7C**(i, ii), **D** (i–iv)]. Among them, cytokines regulated 54% (up) and 46% (down) (**Figure 6A**i), whereas GLT impacted 48% (up) and 52% (down) of significantly expressed transcripts in NS control EndoC-βH3 cells (**Figure 6A**iv). UPF2 KD changed the cytokine-mediated regulation of significantly expressed transcripts by 59% (up) and 41% (down) (**Figure 6A**ii), whereas it did not change the regulation of significantly expressed transcripts by GLT (up: 47%, down: 53%) in EndoC-βH3 cells (**Figure 6A**v). This indicates that UPF2 KD possibly alters the mRNA levels of identified transcript species.

To identify UPF2 KD-regulated transcripts possibly providing protection against cytokine-induced cytotoxicity, we interrogated cytokine- and/or GLT-regulated transcripts that were differentially expressed (*p* < 0.05) in UPF2 KD versus NS control EndoC-βH3 cells using gene enrichment analysis (GEA). Gene Ontology (GO) and Kyoto Encyclopaedia of Genes and Genomes (KEGG) gene enrichment analyses demonstrated that GLT significantly (*p* < 0.05) regulated transcripts in the cellular functions of RNA splicing, mitochondrial inner membrane, and purine nucleoside metabolism [**Supplementary Figure S7E**(i, ii)], although these were not affected by UPF2 KD (**Figure 6B**i). In contrast, GEA revealed that in both untreated and cytokine-treated EndoC-βH3 cells, UPF2 KD significantly regulated transcripts encoding proteins involved in synaptic transmission, extracellular matrix, basolateral plasma membrane, receptor complex, synaptic membrane, transcriptional repressor activity, and enzyme inhibitor activity [**Figures 6B**iii, **C**; **Supplementary Figures S7E**(iii, iv)].

**Figure 7:**
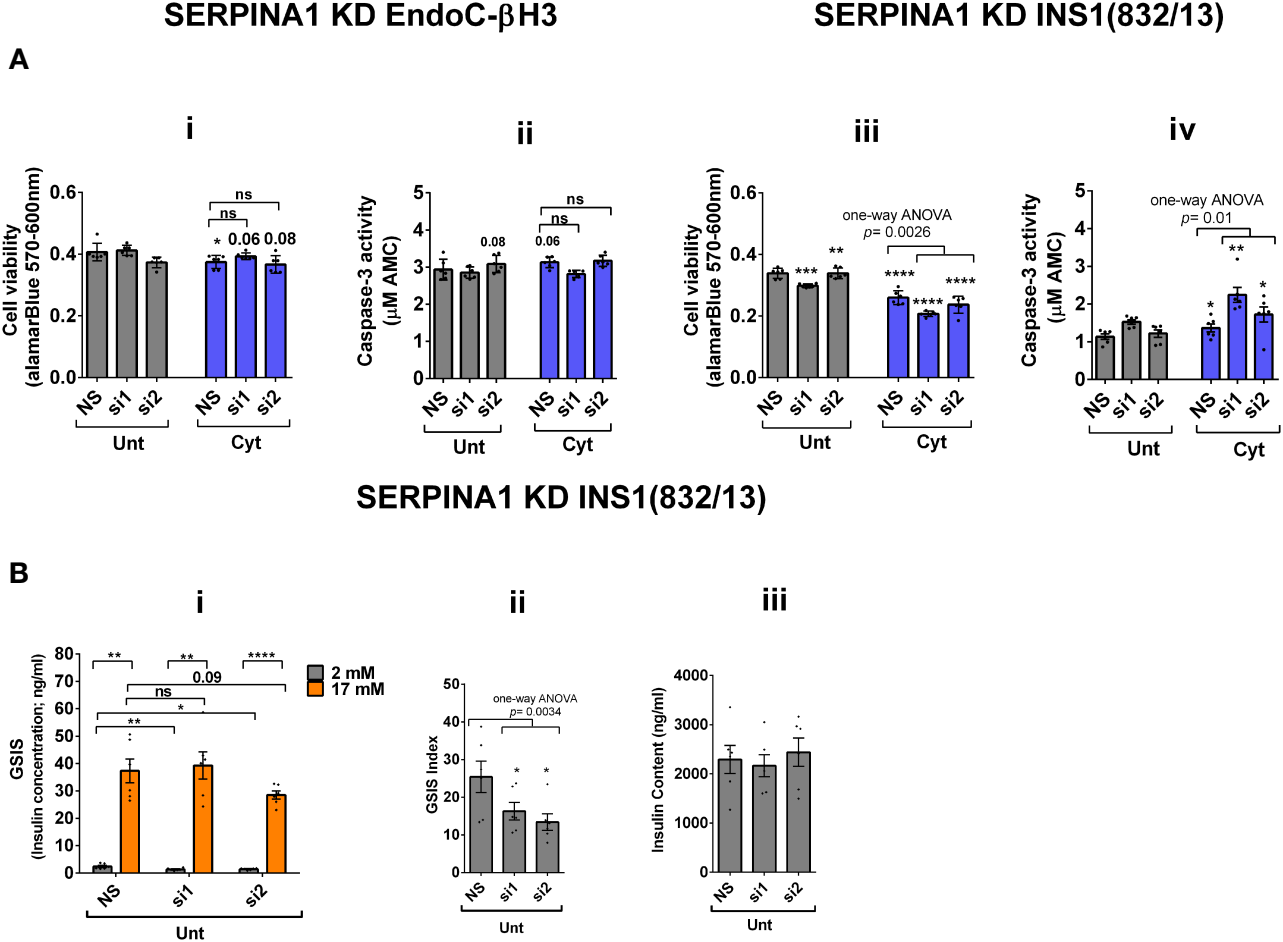
SERPINA1 knockdown deteriorates cytokine cytotoxicity for viability and glucose-stimulated insulin secretion index in INS1(832/13) cells. EndoC-βH3 and INS1(832/13) cells were transfected with siRNAs against SERPINA1 (two species-specific siRNAs for each cell type; si1 and si2) and a nonsilencing siRNA control (NS), incubated for 24 h and exposed to PBS as untreated (Unt) and cytokine combination (Cyt for EndoC-βH3; 3 ng/mL IL-1β + 10 ng/mL IFN-γ+ 10 ng/mL TNF-α) (Cyt for INS1(832/13); 150 pg/mL IL-1β + 0.1 ng/mL IFN-γ+ 0.1 ng/mL TNF-α) for 72 and 24 h, respectively (see **Supplementary Methods**). The knockdown efficiency was checked using quantitative WB [**Supplementary Figures S8**(i–iii)]. **(A)** Cell viability was measured by Alamarblue (i, iii) and caspase-3 activity (ii, iv) assays (*N* = 6). **(B)** Glucose-stimulated insulin secretion (GSIS) (i) and insulin contents (iii) were investigated in the transfected EndoC-βH3 cells. Insulin concentration (ng/mL) was measured by insulin ultrasensitive assay (*N* = 6). GSIS index (ii) was calculated by dividing insulin concentration measured in the treatments of 17 mM by 2 mM glucose. The data are means ± SEM of *N* = 6. The symbol “^*^” indicates the Bonferroni-corrected paired *t*-test values of treatments versus untreated (Unt) NS control or cytokine (Cyt)-treated NS that is, otherwise, designated by a line on top of the bars **(A)**, or corresponding low versus high glucose that is, otherwise, designated by lines on the top of the bars (**B**(i)): ^*^≤ 0.05; ^**^≤ 0.01; ^***^≤ 0.001; ^****^≤ 0.0001. ns, nonsignificant.

RT-qPCR confirmed the logarithmic fold change of UPF2 KD-regulated transcripts in the cytokine-treated EndoC-βH3 cells versus cytokine-treated NS control cells (**Figure 6D**). The role of many of these transcripts in cell viability or insulin secretion was previously identified in pancreatic β cells. Among them, α-1-antitrypsin (1-AT) has been proposed as an antagonist against cytokine-induced pancreatic β-cell death (27, 28). Our expression profiling verified that cytokines upregulated α-1-antitrypsin, and this effect was further potentiated by UPF2 KD. To explore the potential importance of these changes, we knocked down α-1-antitrypsin using specific siRNAs in INS1(832/13) and EndoC-βH3 cells, as confirmed by quantitative WB [**Supplementary Figure S8**(Fig.7B-i, ii and iii)]. The effect of siRNA-mediated α-1-antitrypsin knockdown was inconclusive in EndoC-βH3 cells (**Figure 7A**i, ii) [**Supplementary Figures S7B**(i, ii)]. Both α-1-antitrypsin siRNAs aggravated cytokine-induced cell death in comparison with NS control in INS1(832/13) cells (**Figures 7A**iii, iv). Compared with the NS control, α-1-antitrypsin knockdown reduced the GSIS index but had no effect on insulin content in INS1(832/13) cells (**Figures 7B**i, ii, iii), possibly due to the reduced cell number.

Cytokines reportedly upregulate > 30 splicing factors, affecting alternative splicing of 35% of genes in the human islet transcriptome (6). We examined RNA-seq datasets for alternative splicing (AS) isoforms [**Supplementary Figure S9**(i)] driven by cytokines or GLT versus untreated in the NS control and UPF2 KD EndoC-βH3 cells. Among 2,123 and 2,106 cytokine-driven AS isoforms, skipped exon (SE) isoforms constituted 70.89% (*p* = 0.1, *n* = 6) and 72.5% (*p* = 0.1, *n* = 6) in NS control and UPF2 KD cells, respectively [**Supplementary Figures S9**(ii, iii)]. In contrast, 220 and 133 GLT-driven AS isoforms were identified in NS control and UPF2 KD, respectively [**Supplementary Figures S9**(iv, v)]. This differential regulation could possibly provide a reliable measure for cytokines and the role of GLT in inducing AS isoforms in β cells.

Taken together, the above transcriptome analysis of EndoC-βH3 cells indicates that cytokines increase 1–AT expression, and this is synergised by NMD attenuation.

## Discussion

In this study, we demonstrate that cytokines decrease NMD in INS1(832/13), EndoC-βH3 cells, and dispersed human islets. We also showed that the cytokine-mediated decrease of NMD activity was driven by ER stress and downregulation of UPF3B. Loss-/or gain-of-function of NMD activity could be elicited by UPF3B overexpression or UPF2 knockdown, which led to increases in, or slight decreases in, cytokine-induced apoptosis associated with decreased and increased insulin contents, respectively, without affecting GSIS index in EndoC-βH3 cells. Transcriptome profiling indicated a potentiating effect of UPF2 knockdown on Cyt, but not GLT-mediated, NMD activity. Interestingly, this approach identified transcript targets encoding proteins belonging to the extracellular matrix such as α-1-antitrypsin. Importantly, the knockdown of this gene enhanced cytokine-induced cytotoxicity in β cells.

To the best of our knowledge, the present study represents the first demonstration of a functional effect of cytokines on NMD activity.

UPR activation is known to inhibit NMD via PERK activation and eIF2α phosphorylation to restore IRE1α accumulation and hence a robust UPR activation (19, 20, 29); in addition to the role of PERK activation, our findings suggest that IRE1α riboendonuclease activity (*p* = 0.1) was involved in cytokine-mediated NMD inhibition in EndoC-βH3 cells.

UPF3A and UPF3B act as a potent NMD inhibitors and activators, respectively, in HeLa cells and in mice (23), consistent with our observations following UPF3B overexpression in β cells. However, the finding that forced UPF3A overexpression slightly increased NMD activity in β cells seems inconsistent with previous findings. Recent studies (30, 31) support our apparently discrepant finding regarding the effects of UPF3A overexpression by showing the redundancy of UPF3A and UPF3B as modular activators of NMD (24). With these two earlier studies in mind, we cannot rule out the interference of endogenous UPF3A in the actions of UPF3B on NMD in β cells.

We investigated the consequences of NMD activity on pancreatic β-cell function and viability. The increase in NMD activity by UPF3B overexpression induced basal and cytokine-induced cell death in EndoC-βH3 cells, highlighting the role of increased UPF3B levels in β cells. This appears to be relevant for β-cell viability in both normal and inflammatory stress conditions. Similarly, UPF3B knockdown also caused basal cell death in INS1 cells. Hence, basal UPF3A/B levels seem to play crucial roles in the cell viability of β cells, and perturbation of such a controlled level implicates cell death. On the other hand, the slight protection against cytokine-induced cell death conferred by UPF2 knockdown in EndoC-βH3 cells (**Figures 5A**i, ii) and by SMG6 knockdown in INS1 cells (11) implies a possibly protective mechanism against the cytotoxicity of cytokines in β cells, irrespective to the outcome cytokine-induced cell death. Moreover, our findings provide evidence that the reduced insulin content observed after UPF3B overexpression is related to overactivated NMD.

Cytokine-induced perturbation of NMD (potentiated by UPF2 silencing) might change the balance of anti-/proapoptotic transcripts. This, in turn, may contribute to cytotoxic damage. Consistent with this view, GEA revealed that cytokines deregulate transcripts encoding proteins that localise to and/or function in the extracellular matrix. Thus, α-1-antitrypsin knockdown increased the detachment of MIN6 cells and exacerbated thapsigargin-induced cell death as measured by propidium-iodide staining (28) and, in this study, increased cytokine-induced cell death in INS1(832/13) cells associated with decreased GSIS index.

We speculate that the perturbation of NMD by cytokines leads to increased exon skipping and that this may be part of a feedback loop promoting β-cell plasticity and resilience against cytotoxic cytokines. Future studies will be needed to test this possibility. We note also that depletion of alternative splicing factors (reviewed in (3)) inhibits insulin secretion and induces basal apoptosis after treatment with cytokines in rodent and human β cells (3, 7, 32, 33). Moreover, antisense-mediated exon skipping of 48–50 exons of the dystrophin gene restores the open reading frame and allows the generation of partially to largely functional proteins (34).

In conclusion, we reveal that cytokines suppress NMD activity via ER stress signalling, possibly as a protective response against cytokine-induced NMD component expression. Our findings highlight the central importance of RNA turnover in β-cell responses to inflammatory stress.

### Limitations and future perspectives

We used a luciferase-based NMD reporter based on two separate PTC^−^ and PTC^+^ constructs whose labelled luciferase is separately measured. Thus, a yet-to-develop NMD activity reporter by which transcript RNA, protein, or their corresponding labelled luciferase activity of both PTC^−^ and PTC^+^ transcripts could be examined in one cell rather than (potentially) two separate cells will remove the limitation of the current reporter based on the transfection of the constructs into two separate cells. Moreover, the constant overexpression of UPF3A and UPF3B may result in cell death as β cells cannot cope with the overwhelming levels of these proteins above the basal level. This could explain why UPF3B overexpression reduces the basal cell viability. Further studies should (1) conduct *in vivo* experiments to validate the findings observed *in vitro* and determine if the regulation of NMD in pancreatic β cells is consistent across different cell types and conditions; (2) investigate the mechanisms underlying the regulation of NMD in pancreatic β cells, including the role of specific NMD components and the impact of different stressors on NMD activity; and (3) explore the potential therapeutic implications of targeting NMD in the treatment of inflammatory stress in β cells, including the development of novel drugs or therapies that modulate NMD activity.

### Translatability of the findings

The findings report NMD involvement in the development of islet autoimmunity and the destruction of pancreatic β cells in type 1 diabetes, as well as islet inflammation in type 2 diabetes. The identification of novel targets arising from cytokine-driven NMD attenuation could possibly suggest new biomarkers to monitor disease progression and may also guide the development of protein-based vaccines or antisense mRNA therapeutics in individuals who are at risk of diabetes development and or other inflammatory and autoimmune disorders.

## Data availability statement

The RNA-seq data from the human insulin-producing cell line EndoC-βH3 that support the findings of **Figure 6** of this study are deposited in the Sequence Read Archive (SRA) data repository (Accession numbers for 33 RNA-seq datasets: SRR22938756-SRR22938788) under the BioProject accession number (PRJNA916946) that are appreciated for further citations.

## Ethics statement

Ethical approval was not required for the studies on humans in accordance with the local legislation and institutional requirements because only commercially available established cell lines were used. Ethical approval was not required for the studies on animals in accordance with the local legislation and institutional requirements because only commercially available established cell lines were used. Islet isolation was approved by the Human Research Ethics Board at the University of Alberta (Pro00013094). All donors’ families gave informed consent for the use of pancreatic tissue in research.

## Author contributions

SG: Conceptualization, Formal analysis, Funding acquisition, Investigation, Methodology, Writing – original draft, Writing – review & editing. PM: Funding acquisition, Resources, Writing – original draft, Writing – review & editing. LP: Resources, Writing – original draft, Writing – review & editing. JN: Methodology, Writing – original draft, Writing – review & editing. BP: Investigation, Writing – original draft, Writing – review & editing. TM-P: Writing – original draft, Writing – review & editing. GR: Conceptualization, Funding acquisition, Supervision, Writing – original draft, Writing – review & editing.
